# Bis(2-iodo­thio­phen-3-yl)methanone

**DOI:** 10.1107/S1600536811005472

**Published:** 2011-02-23

**Authors:** Hua Cheng

**Affiliations:** a Department of Chemistry and Biology, Xiangfan University, Xiangfan 441053, People’s Republic of China

## Abstract

In the title mol­ecule, C_9_H_4_I_2_OS_2_, the two five-membered rings form a dihedral angle of 64.2 (2)°. In the crystal, weak inter­molecular C—H⋯O hydrogen bonds link the mol­ecules into layers parallel to the *ab* plane. The crystal packing exhibits short C⋯I contacts of 3.442 (5) Å between the mol­ecules of adjacent layers.

## Related literature

For general background to the synthesis of thio­phene-based conjugated polymers, see: Cheng *et al.* (2009 ▶). For the synthesis of the title compound, see: Brzezinski & Reynolds (2002 ▶).
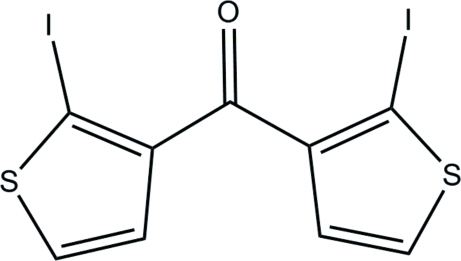


## Experimental

### 

#### Crystal data

C_9_H_4_I_2_OS_2_
*M* *_r_* = 446.04
Monoclinic, 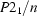

*a* = 10.1908 (9) Å
*b* = 11.4832 (10) Å
*c* = 10.9083 (10) Å
β = 107.600 (1)°
*V* = 1216.77 (19) Å^3^
*Z* = 4
Mo *K*α radiation
μ = 5.48 mm^−1^
*T* = 298 K
0.16 × 0.12 × 0.10 mm

#### Data collection

Bruker SMART CCD area-detector diffractometer
Absorption correction: multi-scan (*SADABS*; Sheldrick, 1996 ▶) *T*_min_ = 0.474, *T*_max_ = 0.610
8022 measured reflections
3003 independent reflections
2541 reflections with *I* > 2σ(*I*)
*R*_int_ = 0.084

#### Refinement

*R*[*F* ^2^ > 2σ(*F* ^2^)] = 0.043
*wR*(*F* ^2^) = 0.103
*S* = 1.11
3003 reflections
127 parameters
H-atom parameters constrained
Δρ_max_ = 1.08 e Å^−3^
Δρ_min_ = −0.67 e Å^−3^

### 

Data collection: *SMART* (Bruker, 2001 ▶); cell refinement: *SAINT* (Bruker, 1999 ▶); data reduction: *SAINT*; program(s) used to solve structure: *SHELXS97* (Sheldrick, 2008 ▶); program(s) used to refine structure: *SHELXL97* (Sheldrick, 2008 ▶); molecular graphics: *SHELXTL* (Sheldrick, 2008 ▶); software used to prepare material for publication: *SHELXTL*.

## Supplementary Material

Crystal structure: contains datablocks I, global. DOI: 10.1107/S1600536811005472/cv5050sup1.cif

Structure factors: contains datablocks I. DOI: 10.1107/S1600536811005472/cv5050Isup2.hkl

Additional supplementary materials:  crystallographic information; 3D view; checkCIF report

## Figures and Tables

**Table 1:** Hydrogen-bond geometry (Å, °)

*D*—H⋯*A*	*D*—H	H⋯*A*	*D*⋯*A*	*D*—H⋯*A*
C4—H4⋯O1^i^	0.93	2.51	3.233 (7)	135
C8—H8⋯O1^ii^	0.93	2.40	3.324 (6)	172

Symmetry codes: (i) 
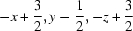
; (ii) 
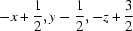
.

**Fig. 1.**
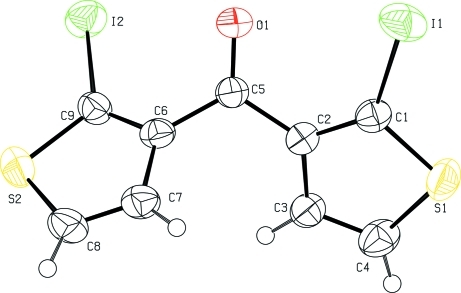
The title molecule with the atom-numbering scheme. The displacement ellipsoids are drawn at the 50% probability level.

## supplementary crystallographic information

### Comment

The title compound, (I),  is an important organic intermediate for the synthesis of conjugated polymers for organic solar cell applications (Brzezinski & Reynolds, 2002; Cheng *et al.*, 2009). In (I) (Fig. 1), two five-membered rings form a dihedral angle of 64.2 (2)°. Weak intermolecular C—H···O hydrogen bonds link the molecules into layers parallel to *ab* plane. The crystal packing exhibits short C···I contacts of 3.442 (5) Å between the molecules from the neighbouring layers.

### Experimental

The title compound was synthesized according to the reported method by Brzezinski & Reynolds (2002). After being dissolved in the mixture of MeOH-Hexane (1:3) for seversal days, colourless crystals suitable for single-crystal X-ray diffraction were obtained.

### Refinement

All hydrogen atoms were positioned geometrically (C—H = 0.93 Å) and allowed to ride on their parent atoms, with *U*_iso_(H) = 1.2 *U*_eq_(C).

### Crystal data

**Table:**

C_9_H_4_I_2_OS_2_	*F*(000) = 816
*M**_r_* = 446.04	*D*_x_ = 2.435 Mg m^−^^3^
Monoclinic, *P*2_1_/*n*	Mo *K*α radiation, λ = 0.71073 Å
Hall symbol: -P 2yn	Cell parameters from 2906 reflections
*a* = 10.1908 (9) Å	θ = 2.4–26.9°
*b* = 11.4832 (10) Å	µ = 5.48 mm^−^^1^
*c* = 10.9083 (10) Å	*T* = 298 K
β = 107.600 (1)°	Block, colourless
*V* = 1216.77 (19) Å^3^	0.16 × 0.12 × 0.10 mm
*Z* = 4	

### Data collection

**Table:**

Bruker SMART CCD area-detector diffractometer	3003 independent reflections
Radiation source: fine-focus sealed tube	2541 reflections with *I* > 2σ(*I*)
graphite	*R*_int_ = 0.084
φ and ω scans	θ_max_ = 28.2°, θ_min_ = 2.4°
Absorption correction: multi-scan (*SADABS*; Sheldrick, 1996)	*h* = −13→7
*T*_min_ = 0.474, *T*_max_ = 0.610	*k* = −15→12
8022 measured reflections	*l* = −14→14

### Refinement

**Table:**

Refinement on *F*^2^	Primary atom site location: structure-invariant direct methods
Least-squares matrix: full	Secondary atom site location: difference Fourier map
*R*[*F*^2^ > 2σ(*F*^2^)] = 0.043	Hydrogen site location: inferred from neighbouring sites
*wR*(*F*^2^) = 0.103	H-atom parameters constrained
*S* = 1.11	*w* = 1/[σ^2^(*F*_o_^2^) + (0.0342*P*)^2^] where *P* = (*F*_o_^2^ + 2*F*_c_^2^)/3
3003 reflections	(Δ/σ)_max_ = 0.001
127 parameters	Δρ_max_ = 1.08 e Å^−^^3^
0 restraints	Δρ_min_ = −0.67 e Å^−^^3^

### Special details

**Table:**

Geometry. All e.s.d.'s (except the e.s.d. in the dihedral angle between two l.s. planes) are estimated using the full covariance matrix. The cell e.s.d.'s are taken into account individually in the estimation of e.s.d.'s in distances, angles and torsion angles; correlations between e.s.d.'s in cell parameters are only used when they are defined by crystal symmetry. An approximate (isotropic) treatment of cell e.s.d.'s is used for estimating e.s.d.'s involving l.s. planes.
Refinement. Refinement of *F*^2^ against ALL reflections. The weighted *R*-factor *wR* and goodness of fit *S* are based on *F*^2^, conventional *R*-factors *R* are based on *F*, with *F* set to zero for negative *F*^2^. The threshold expression of *F*^2^ > σ(*F*^2^) is used only for calculating *R*-factors(gt) *etc*. and is not relevant to the choice of reflections for refinement. *R*-factors based on *F*^2^ are statistically about twice as large as those based on *F*, and *R*- factors based on ALL data will be even larger.

### Fractional atomic coordinates and isotropic or equivalent isotropic displacement parameters (Å^2^)

**Table:**

	*x*	*y*	*z*	*U*_iso_*/*U*_eq_	
C1	0.7428 (5)	0.3871 (4)	0.9017 (5)	0.0377 (10)	
C2	0.6198 (5)	0.3657 (4)	0.8086 (4)	0.0328 (9)	
C3	0.6258 (5)	0.2588 (4)	0.7431 (5)	0.0392 (11)	
H3	0.5515	0.2291	0.6783	0.047*	
C4	0.7482 (6)	0.2053 (5)	0.7835 (5)	0.0486 (13)	
H4	0.7688	0.1359	0.7495	0.058*	
C5	0.5029 (5)	0.4469 (4)	0.7705 (5)	0.0349 (10)	
C6	0.3643 (5)	0.3977 (4)	0.7066 (5)	0.0350 (10)	
C7	0.3112 (6)	0.2950 (4)	0.7492 (5)	0.0438 (12)	
H7	0.3628	0.2488	0.8166	0.053*	
C8	0.1801 (6)	0.2717 (5)	0.6827 (6)	0.0499 (13)	
H8	0.1300	0.2093	0.6996	0.060*	
C9	0.2680 (5)	0.4481 (4)	0.6055 (5)	0.0372 (10)	
I1	0.79608 (5)	0.51950 (4)	1.03536 (4)	0.05838 (16)	
I2	0.28839 (4)	0.58784 (3)	0.49276 (4)	0.05046 (14)	
O1	0.5191 (4)	0.5512 (3)	0.7870 (5)	0.0569 (10)	
S1	0.86152 (15)	0.28059 (13)	0.90534 (14)	0.0517 (4)	
S2	0.11662 (14)	0.37150 (14)	0.56270 (15)	0.0512 (3)	

### Atomic displacement parameters (Å^2^)

**Table:**

	*U*^11^	*U*^22^	*U*^33^	*U*^12^	*U*^13^	*U*^23^
C1	0.043 (3)	0.035 (3)	0.034 (2)	0.002 (2)	0.012 (2)	0.0020 (19)
C2	0.036 (3)	0.033 (2)	0.030 (2)	0.0025 (19)	0.011 (2)	0.0009 (18)
C3	0.043 (3)	0.037 (3)	0.034 (2)	0.004 (2)	0.006 (2)	−0.003 (2)
C4	0.059 (4)	0.044 (3)	0.042 (3)	0.010 (2)	0.012 (3)	−0.006 (2)
C5	0.034 (2)	0.030 (2)	0.043 (3)	0.0002 (18)	0.014 (2)	0.000 (2)
C6	0.039 (3)	0.028 (2)	0.042 (2)	0.0007 (18)	0.018 (2)	−0.0017 (19)
C7	0.049 (3)	0.036 (3)	0.047 (3)	−0.002 (2)	0.014 (2)	0.006 (2)
C8	0.048 (3)	0.040 (3)	0.066 (4)	−0.011 (2)	0.023 (3)	0.003 (3)
C9	0.035 (3)	0.035 (2)	0.043 (3)	−0.0045 (19)	0.014 (2)	−0.002 (2)
I1	0.0753 (3)	0.0496 (2)	0.0436 (2)	−0.01260 (18)	0.0080 (2)	−0.01168 (16)
I2	0.0553 (3)	0.0435 (2)	0.0549 (2)	0.00141 (15)	0.02021 (19)	0.01160 (16)
O1	0.041 (2)	0.0288 (18)	0.094 (3)	0.0001 (15)	0.011 (2)	−0.005 (2)
S1	0.0430 (8)	0.0537 (9)	0.0501 (8)	0.0140 (6)	0.0018 (6)	0.0014 (6)
S2	0.0357 (7)	0.0542 (8)	0.0600 (8)	−0.0072 (6)	0.0092 (6)	0.0008 (7)

### Geometric parameters (Å, °)

**Table:**

C1—C2	1.376 (7)	C5—C6	1.485 (7)
C1—S1	1.712 (5)	C6—C9	1.364 (7)
C1—I1	2.063 (5)	C6—C7	1.433 (6)
C2—C3	1.431 (7)	C7—C8	1.339 (8)
C2—C5	1.471 (7)	C7—H7	0.9300
C3—C4	1.339 (8)	C8—S2	1.713 (6)
C3—H3	0.9300	C8—H8	0.9300
C4—S1	1.709 (6)	C9—S2	1.714 (5)
C4—H4	0.9300	C9—I2	2.070 (5)
C5—O1	1.215 (6)		
			
C2—C1—S1	111.7 (4)	C9—C6—C7	111.2 (4)
C2—C1—I1	129.8 (4)	C9—C6—C5	124.6 (4)
S1—C1—I1	118.5 (3)	C7—C6—C5	124.1 (4)
C1—C2—C3	110.8 (4)	C8—C7—C6	113.7 (5)
C1—C2—C5	125.1 (4)	C8—C7—H7	123.2
C3—C2—C5	123.8 (4)	C6—C7—H7	123.2
C4—C3—C2	113.9 (5)	C7—C8—S2	111.4 (4)
C4—C3—H3	123.1	C7—C8—H8	124.3
C2—C3—H3	123.1	S2—C8—H8	124.3
C3—C4—S1	111.6 (4)	C6—C9—S2	111.8 (4)
C3—C4—H4	124.2	C6—C9—I2	129.3 (4)
S1—C4—H4	124.2	S2—C9—I2	118.7 (3)
O1—C5—C2	121.4 (4)	C4—S1—C1	92.1 (3)
O1—C5—C6	120.8 (4)	C8—S2—C9	92.0 (3)
C2—C5—C6	117.8 (4)		
			
S1—C1—C2—C3	1.2 (5)	C2—C5—C6—C7	44.7 (7)
I1—C1—C2—C3	−175.7 (4)	C9—C6—C7—C8	−0.8 (6)
S1—C1—C2—C5	−172.4 (4)	C5—C6—C7—C8	175.3 (5)
I1—C1—C2—C5	10.6 (7)	C6—C7—C8—S2	1.6 (6)
C1—C2—C3—C4	−1.6 (6)	C7—C6—C9—S2	−0.3 (5)
C5—C2—C3—C4	172.2 (5)	C5—C6—C9—S2	−176.4 (4)
C2—C3—C4—S1	1.2 (6)	C7—C6—C9—I2	−173.7 (4)
C1—C2—C5—O1	24.2 (7)	C5—C6—C9—I2	10.1 (7)
C3—C2—C5—O1	−148.6 (5)	C3—C4—S1—C1	−0.4 (5)
C1—C2—C5—C6	−158.0 (5)	C2—C1—S1—C4	−0.5 (4)
C3—C2—C5—C6	29.2 (7)	I1—C1—S1—C4	176.8 (3)
O1—C5—C6—C9	38.0 (7)	C7—C8—S2—C9	−1.5 (5)
C2—C5—C6—C9	−139.7 (5)	C6—C9—S2—C8	1.0 (4)
O1—C5—C6—C7	−137.6 (5)	I2—C9—S2—C8	175.2 (3)

### Hydrogen-bond geometry (Å, °)

**Table:**

*D*—H···*A*	*D*—H	H···*A*	*D*···*A*	*D*—H···*A*
C4—H4···O1^i^	0.93	2.51	3.233 (7)	135
C8—H8···O1^ii^	0.93	2.40	3.324 (6)	172

Symmetry codes: (i) −*x*+3/2, *y*−1/2, −*z*+3/2; (ii) −*x*+1/2, *y*−1/2, −*z*+3/2.
